# Boosting Tumor-Specific Immunity Using PDT

**DOI:** 10.3390/cancers8100091

**Published:** 2016-10-06

**Authors:** Nicole Maeding, Thomas Verwanger, Barbara Krammer

**Affiliations:** Division of Molecular Tumor Biology, Department of Molecular Biology, University of Salzburg, Hellbrunnerstrasse 34, 5020 Salzburg, Austria; nicole.maeding.hh@gmail.com (N.M.); thomas.verwanger@sbg.ac.at (T.V.)

**Keywords:** photodynamic tumor therapy, tumor-specific immunity, antitumor immunity, regulatory T cells, dendritic cells, memory

## Abstract

Photodynamic therapy (PDT) is a cancer treatment with a long-standing history. It employs the application of nontoxic components, namely a light-sensitive photosensitizer and visible light, to generate reactive oxygen species (ROS). These ROS lead to tumor cell destruction, which is accompanied by the induction of an acute inflammatory response. This inflammatory process sends a danger signal to the innate immune system, which results in activation of specific cell types and release of additional inflammatory mediators. Activation of the innate immune response is necessary for subsequent induction of the adaptive arm of the immune system. This includes the priming of tumor-specific cytotoxic T lymphocytes (CTL) that have the capability to directly recognize and kill cells which display an altered self. The past decades have brought increasing appreciation for the importance of the generation of an adaptive immune response for long-term tumor control and induction of immune memory to combat recurrent disease. This has led to considerable effort to elucidate the immune effects PDT treatment elicits. In this review we deal with the progress which has been made during the past 20 years in uncovering the role of PDT in the induction of the tumor-specific immune response, with special emphasis on adaptive immunity.

## 1. Introduction

Photodynamic therapy (PDT) is a cancer treatment modality, for which the principle had already been proposed over a century ago [1]. It is an alternative treatment among the currently available therapies that offers minimal side effects for the patient while maintaining high efficiency [2]. As a clinically approved therapy it is used for the treatment of early staged disease and superficial cancer types, and as palliative easement in terminal/late staged cancers [3,4]. PDT utilizes a light-sensitive photosensitizer (PS), which is applied systemically or locally, and visible light of appropriate wavelengths to excite the PS. After tumor-selective accumulation, the photosensitizer is locally photoactivated by nonthermal light irradiation; subsequently it either emits fluorescence light (diagnostic use), or reacts with surrounding molecules. In the presence of molecular oxygen, reactive oxygen species (ROS) are formed, which oxidize proteins and lipids in the target cells. This leads to stimulation of signaling processes as well as target destruction by apoptotic and necrotic cell death (therapeutic use) [3,5]. Indirect effects leading to tumor cell destruction include vascular shutdown by damaging endothelial cells and the vascular basement membrane. This results in blood flow stasis, tissue hemorrhages, and oxygen deprivation. Furthermore, induction of an inflammatory response and immune reactions directed against the tumor contribute to fighting off primary as well as secondary disease manifestations [6]. However, PDT still constitutes more of a fringe option than a regular treatment for cancer patients. This might be attributed to several limitations PDT faces: obviously, applicability is restricted by tumor site accessibility and penetration depth of light needed to excite the photosensitizer. This limits therapeutic success, mainly to outer and inner body surfaces and flat tumors. In addition, classical PDT is only suited for solid tumors. Tumor cells circulating in the vasculature cannot be treated using traditional PDT protocols. During the past years, efforts have been made to develop advanced protocols that overcome these disadvantages. Interstitial PDT using optical fibers as light source and self-illuminated PDT show promising results for the treatment of solid tumors of larger size (as compared to flat tumors) and for solid lesions in parenchymal organs. For eradication of circulating tumor cells, efforts are being made to develop extracorporeal PDT protocols which employ an antibody-conjugated PS to target specific cells, and illumination of the blood is conducted afterwards [7,8,9]. Most advantageous—and currently under extensive research—is the induction of antitumor immune reactions by PDT. These reactions serve to support primary tumor elimination and to extend the local antitumor response to systemic surveillance to combat disease recurrence, metastases, or circulating tumor cells [10]. This has even led to several approaches to use PDT as vaccine [11,12]. In this review we focus on a collection of findings related to PDT-mediated tumor-specific immunity and their implications for future directions in the field of photodynamic tumor therapy.

## 2. PDT and Innate Immunity

The local trauma inflicted by PDT treatment on the tumor cells, the vasculature, and the surrounding tissue causes induction and release of various mediators leading to an inflammatory reaction to initiate an immune response. The oxidative stress due to the excessive generation of ROS results in surface expression and secretion of damage-associated molecular patterns (DAMPs) as well as inflammatory mediators which are released from dying and damaged cells. DAMPs are molecules derived from host cells to signal cell injury or death. They predominantly comprise nuclear or cytosolic proteins which become released from the cell or exposed on its surface and serve in the initiation of a noninfectious immune response. Recognition of DAMPs via engagement with their respective receptors on infiltrating immune cells (so-called pattern recognition receptors, PRRs) aids in signaling the nature of the underlying threat to the immune system and enabling the appropriate immune response. DAMPs reported to be necessary for the generation of antitumor immunity and induced upon PDT include surface calreticulin (CRT), heat shock protein (HSP) 70, HSP90, ATP, and high-mobility group box 1 protein (HMGB1) [13,14]. Inflammatory mediators include cytokines and chemokines. Cytokines are small, secreted proteins produced mostly by immune cells, but also by endothelial and stromal cells as well as fibroblasts. Their main function is to promote or inhibit proliferation, activation, and differentiation of immune cells, thus they are commonly divided into proinflammatory and anti-inflammatory or immunosuppressive cytokines. Prominent examples for proinflammatory cytokines are interleukin (IL)-12 and IL-4, which are necessary for the differentiation of T helper cells type 1 (Th1) and type 2 (Th2), respectively. Classical anti-inflammatory or immunosuppressive cytokines include IL-10 and transforming growth factor (TGF)-β. IL-10 effectively inhibits expression of Th1 cytokines and major histocompatibility complex (MHC) II and macrophage activation. TGF-β inhibits cell proliferation and induces differentiation of regulatory T cells (Treg), an immunosuppressive subtype of T helper cells. Chemokines are small cytokines which build up gradients in the affected area and serve as chemoattractants. They are essential for directing the migration and activation of phagocytes and lymphocytes in the course of an inflammatory reaction. Guided by chemotactic gradients, inflammatory immune cells enter the affected region to launch an immune reaction and remove the source of the threat. 

### 2.1. Cytokine Release

Elevated levels of a variety of cytokines have been shown in animal as well as human studies. Increased production of IL-6 appears to be a frequent event after PDT [15,16,17,18,19]. IL-6 is considered to be a proinflammatory cytokine which stimulates the immune response, the induction of fever, and acute-phase proteins. However, results on the impact or function of IL-6 in PDT outcome differ. In a system of EMT6 tumors treated with Photofrin^®^-PDT, blocking of IL-6 with respective antibodies significantly reduced PDT-induced neutrophilia at 2 hours and 8 hours post-treatment [20]. Contrasting this, another study found no effect of anti-IL-6 treatment on intratumoral neutrophil levels after PDT [16]. Those contradictory findings may be attributed to the different photosensitizers used, evaluation of blood neutrophil levels vs intratumoral neutrophil numbers, and different treatment protocols and evaluation time points. Another prominent cytokine elevated after PDT is IL-1β. In a model of rat rhabdomyosarcoma it was shown that increased IL-1β preceded PDT-induced neutrophilia [21]. In subsequent studies, other groups were able to demonstrate that IL-1β is indeed a crucial mediator in PDT outcome and neutrophilia since blocking of this cytokine led to a significantly decreased rate of tumor cures and neutrophils in the tumor-draining lymph nodes (TDLN) in response to treatment [22,23]. Other cytokines that have been reported to be elevated post PDT include tumor necrosis factor (TNF)-α and interferon (IFN)γ [10]. The chemokines CXCL1 (chemokine (C-X-C) ligand 1 or keratinocyte chemoattractant, KC) and CXCL2 (macrophage inflammatory protein-2, MIP-2) were also shown to be increased after PDT treatment in a murine model of EMT6 carcinoma. These two chemokines are known for possessing neutrophil chemoattractant activity. However, only CXCL2 was shown to be necessary for neutrophil migration into the tumors in this setting [16]. In a rather recent report, Brackett et al. found induction of the cytokine IL-17 after treatment. This cytokine proved its importance by acting upstream of IL-1β to regulate its expression levels. Thereby, it indirectly controlled the expression of CXCL2 which was dependent on IL-1β. Diminished levels were found for TGF-β in the sera of CT26 colon carcinoma-bearing mice after PDT treatment with benzoporphyrin derivative (BPD) [24]. Additionally, blockade of immunosuppressive cytokines TGF-β and IL-10 has been shown to greatly enhance PDT-mediated tumor cure rates in C3H/HeN mice with subcutaneous FsaR fibrosarcomas [25]. 

### 2.2. Neutrophils

The importance of neutrophils in PDT efficiency has been proven in numerous studies. Neutrophils pose the first line of defense against pathogens and inflammatory insults to the host. In order to do so they secrete leukotrienes, prostaglandins, and cytokines to initiate the development of the inflammatory response. Furthermore, they are able to directly kill pathogens and they have been reported to be able to present antigens via MHC class II under certain circumstances. This raises the possibility that neutrophils could aid in the activation of CD4^+^ T helper cells. Increased levels of neutrophils following PDT have been reported frequently [22,23,26,27,28]. Moreover, Korbelik et al. showed that depletion of neutrophils led to a drop in mice cured from EMT6 tumors to 30%, with tumors recurring after 2–3 weeks [29]. In line with this are findings from Kousis and coworkers demonstrating that neutrophil depletion resulted in diminished numbers of activated cytotoxic T lymphocytes (CTL) in the TDLN and the tumor tissue [30]. In a different approach using blockade of neutrophil migration by administering anti-ICAM1 (intercellular adhesion molecule 1) the group of Sun demonstrated complete abrogation of the curative outcome of their treatment regimen. Additionally, this work showed an impairment of the curative effect following administration of anti-IL-1β [22]. This finding was further highlighted by studies from Brackett et al. [23]. In their setting, neutrophil entry into TDLN post-PDT was mediated by CXCR2–CXCL2 interaction, with CXCL2 induction being dependent on IL-17A and IL-1β. Interestingly, the degree of neutrophil infiltration appears to be governed by the treatment regimen applied. Work from Shams et al. revealed that the highest degree of neutrophil influx into tumor tissue and TDLN was achieved with a treatment regimen delivering a low fluence at a low fluence rate. This was further accompanied by a substantial increase in activated CTL [31]. A previous study already demonstrated the highest degree of induction of proinflammatory cytokines IL-6, MIP-1, and MIP-2 under these treatment conditions compared to regimens delivered at higher fluence and/or higher fluence rate [19]. 

### 2.3. Dendritic Cells

Crucial for the induction of an adaptive immune response are dendritic cells (DCs). DCs are professional antigen-presenting cells (APC) and as such their main function is to present endogenous (e.g., viral) as well as exogenous (e.g., bacterial) antigens to lymphocytes in order to activate them and mount an appropriate immune response. They exist in two functionally distinct stages, “mature” and “immature”. Immature DCs constantly sample their environment by taking up antigens via macropinocytosis, receptor-mediated endocytosis, and phagocytosis. They are characterized by expression of CD11c and low levels of MHC I, MHC II, CD80, and CD86 and the relative absence of cytokine production. In the presence of inflammatory stimuli, those immature DCs differentiate into their mature state. This includes upregulation of processing and presentation of antigens and increased expression of MHC molecules and the costimulatory molecules CD80 and CD86. Additionally, maturation induces secretion of proinflammatory cytokines including IL-12, IL-6, and IL-1β. Mature DCs migrate to the lymph nodes in large numbers where they present peptide–MHC complexes to lymphocytes. In combination with appropriate costimulation, this leads to activation of CD4^+^ T helper cells, CD8^+^ CTL, and B cells, and initiates the adaptive immune response [32,33]. 

Several groups have shown the importance of DCs for PDT-mediated antitumor immunity and efficiency. Jalili and coworkers were able to demonstrate that intratumoral injection of immature DCs after PDT treatment resulted in significantly delayed tumor growth of the treated tumor and of untreated tumors in the contralateral hind limb [34]. Similar outcome was reported by the group of Saji [35]. Further corroborating these findings were experiments by Preise et al.: DTR bone marrow chimera mice were inoculated with CT26 colon carcinoma cells, and subcutaneously growing tumors were subjected to PDT. Depletion of DCs by injection of diphtheria toxin (DTx) resulted in increased recurrence rates of the tumors. Mice which were systemically depleted of DCs showed 90% of disease recurrence compared to only 20% in mice which received PDT treatment only [36]. Furthermore, other reports support the involvement of DCs in the response to PDT as evidenced by enhanced maturation and activation as well as increased secretion of proinflammatory cytokines after treatment [37,38,39,40].

## 3. PDT and Adaptive Immunity

The first evidence for induction of a tumor-specific immune response following PDT came from Canti et al. in 1994. This group demonstrated that normal mice cured from MS-2 fibrosarcoma by PDT were able to resist a rechallenge with tumor cells in a tumor-specific manner. In contrast to this, immunosuppressed counterparts were not able to resist the rechallenge [41]. Since then, several studies have shown that an intact immune system—specifically the adaptive arm of the immune response—is crucial for PDT outcome. Korbelik et al. demonstrated that treatment of BALB/c mice bearing EMT6 mammary carcinomas with PDT resulted in complete cures, whereas SCID mice did not elicit the same antitumor response with the identical PDT regimen [27]. Since SCID mice lack mature T lymphocytes, they are not able to mount a cellular adaptive immune response. Likewise, the group of Preise successfully treated BALB/c mice with a vascular-targeted PDT approach from CT26 tumors with cure rates of more than 70%. When the same experiments were carried out in BALB/nude or SCID mice, cure rates dropped to 18% and 11%, respectively [36]. Similar findings were repeatedly reported from other groups as well over the years [10,42,43]. 

Induction of systemic and memory immunity following PDT treatment has also been verified in numerous studies. Systemic immunity is reflected by the extension of the locally induced immune response to distant nontreated areas. A study by Kabingu and coworkers demonstrated the destruction of lung metastases indicative of an ongoing systemic immune response: mice were inoculated subcutaneously (s.c.) and intravenously (i.v.) with EMT6 tumor cells to generate a primary tumor (s.c.) and lung tumors mimicking metastases (i.v.). Treatment of the primary tumor with Photofrin^®^–PDT led to 90%–100% of tumor ablation, and analysis of lung metastases 10 days after PDT revealed a significant reduction of lung tumors compared to nontreated controls [42]. Likewise, Mroz et al. reported regression of distant untreated tumors. In their model, mice bearing bilateral s.c. CT26.CL25 tumors were treated with BPD–PDT on one side while the contralateral tumor was left untreated. In a total of seven out of nine mice, this approach led to complete and permanent regression of the contralateral tumor [10]. Recent publications by other groups further support those findings [31,43]. Additionally, clinical observations report the regression of lesions outside the treatment field after PDT-treatment as well, thus indicating the generation of systemic immunity in human patients [44,45,46]. 

Recurring disease can only be prevented when memory immunity is successfully generated. In experimental models of cancer, generation of such a memory immunity can be evaluated by resistance of previously cured subjects to rechallenge with the same type of cancer cells. The first study indicating the induction of tumor-specific immune memory was published by Korbelik et al. in 1999: this work showed the ability of SCID mice to resist tumor cell rechallenge after they were cured from EMT6 tumors by a combination approach of adoptive transfer of tumor-sensitized splenocytes and PDT treatment [47]. The group of Preise demonstrated long-lasting protection against rechallenge in immunocompetent mice cured from primary tumors using a vascular-targeted approach of PDT. Interestingly, this approach even resulted in partial cross-protection against a different type of tumor cell used for rechallenge. However, the mechanism underlying this observation still remains to be elucidated [36]. In another study by Sanovic et al., BALB/c mice bearing a s.c. CT26 colon carcinoma were treated with hypericin–PDT, and this treatment yielded a striking 100% of tumor cures, which lasted until the end of the study. Additionally, i.v. challenge of those cured mice with viable CT26 cells showed no development of new tumors, thereby indicating existence of systemic memory immunity. Notably, these results were obtained with a protocol using a low PS dose delivered at a low fluence and low fluence rate [48]. Similar findings were reported by Mroz et al. who demonstrated 95% of cured mice resisting tumor development upon subsequent rechallenge [10]. Reginato and coworkers achieved 90% of cures with a treatment protocol employing BPD and Treg depletion using cyclophosphamide in a CT26 tumor model. Sixty-five percent of these mice rejected the rechallenge. However, with this protocol, another round of Treg depletion prior to rechallenge was necessary to unravel the memory immunity [24]. Interestingly, this group used the same type of tumor model (CT26 colon carcinoma in BALB/c mice) as Sanovic et al., whose studies showed no requirement for Treg depletion for therapeutic success. These differences might be attributed to the use of different PS (hypericin vs BPD) and/or differences in fluence and fluence rate (14 J cm^−2^@27 mW cm^−2^ vs. 120 J cm^−2^@100 mW cm^−2^). Other groups assessed induction of memory immunity by resistance to rechallenge as well and reported results in line with the abovementioned findings [31,43]. 

The mediators of the adaptive immune response are antigen-specific B and T cells. Upon antigen recognition, B cells produce the antibodies necessary for eliminating extracellular pathogens. Although there are pieces of data pointing to a role for B cells in PDT-induced antitumor immunity [27,36,43], the importance of this humoral response has remained largely uninvestigated. In contrast to that, considerable efforts have been made over the past two decades to elucidate the role of the cellular immune response, i.e., the role of T lymphocytes. The findings regarding this population will be discussed in the following sections. 

### 3.1. CD4^+^ T Cells

T cells are divided into several subpopulations. When activated, CD4^+^ T helper cells differentiate into distinct lineages, a process which is dependent on the stimuli and cytokines present. Those lineages secrete defined cytokines to assist in clearance of intracellular pathogens and extracellular microorganisms. Furthermore, CD4^+^ T helper cells provide help for B cells and CD8^+^ cytotoxic T cells for their activation and the generation of memory cells. The best defined T helper cell populations are Th1, Th2, and Th17. Th2 cells and their associated cytokines—IL-4, IL-5, and IL-13—serve in the response to extracellular microorganisms and the induction of the B cell isotype switching. Th1 cells are characterized by production of IFNγ and are crucial for the eradication of intracellular pathogens. IFNγ is known to activate the bactericidal activity of macrophages and increase the expression of MHC I on normal cells and MHC II on APCs. Thereby it facilitates processing and presentation of endogenous antigens, which makes infected and aberrant cells visible to the immune system. Furthermore, IFNγ promotes activity of natural killer (NK) cells, which recognize and eliminate cells with decreased MHC I expression. Th1 cells have implications in antitumor immunity via activation and regulation of CTL, cross-priming of the immune response by APCs (this enables presentation of intracellular antigens in the context of MHC II), and direct tumor cell-killing through the release of specific cytokines [49]. The third subtype of T helper cells, that is well established by now, are Th17 cells and their signature cytokine IL-17. IL-17 leads to stimulation and de novo generation of neutrophils and is important in the response to certain extracellular bacteria and fungi [50]. In cancer, Th17 cells seem to be able to have opposing roles. There are numerous reports showing eradication of tumors by Th17 cells and a beneficial effect of their abundance in the tumor microenvironment. However, an equally solid body of evidence suggests a role for this subpopulation in tumor progression. This opposing impact of Th17 cells on tumor immunity seems to be attributable to the fact that the fine-tuning of their differentiation and function is highly dependent on a variety of factors; these include the type of tumor, the composition of stimuli leading to their activation (e.g., cytokine composition, T cell receptor signaling strength) and the therapeutic approach applied [51]. Interestingly, Th17 cells share ties with immunosuppressive Treg. In a proinflammatory environment, the decision of whether naïve T cells develop into either Treg or Th17 is dependent on the amount of available TGF-β: at low concentrations it synergizes with IL-6 and/or IL-21 and drives the induction of Th17 cells. At high TGF-β concentrations these cytokines are no longer sufficient to overcome Foxp3 (forkhead box protein 3)-mediated repression of retinoic acid receptor (RAR)-related orphan receptor (ROR)γt, and the cells differentiate into Treg [52]. Additionally, Th17 cells are able to acquire a Th1-like phenotype with the ability to secrete IFNγ when they are exposed to IL-12 [51]. The role of T helper cells in PDT-mediated immunity is somewhat controversial. Experiments by Kabingu et al. showed no effect of CD4^+^ T cell depletion on PDT efficiency and induction of systemic antitumor immunity [42]. In contrast to that, other groups found a dependency of treatment outcome on the presence of CD4^+^ T cells. The group of Korbelik used antibodies for CD4, CD25, and a combination of both to deplete T helper cells, and saw a drop in cures by 30%–50% [29,47]. In line with this are results from others reporting delayed or abrogated tumor growth in naïve mice following adoptive transfer of CD4^+^ T cells from PDT-cured mice. Further analysis of this CD4^+^ population showed increased IFNγ secretion upon restimulation, which is indicative for the generation of a Th1 response [36]. However, the precise mechanisms by which CD4^+^ T cells contribute to PDT outcome remain largely elusive so far. Some light on this was shed by Brackett and coworkers, who showed an increase of Th17 cells and the corresponding signature cytokine IL-17A in the tumor-draining lymph nodes after PDT treatment [23]. In a very recent work Garg et al. found elevated levels of Th1 and Th17 cells in the brain immune contexture of mice treated with an immunogenic cell death (ICD)-based DC vaccine against high-grade glioma (HGG) and subsequently inoculated with the respective glioma cells. Furthermore, splenic T cells from these mice exhibited higher IFNγ production upon restimulation with naïve GL261 cell lysates, thus indicating expansion of localized immunostimulation in the brain to a systemic effect crucial for long-term immunity [14].

### 3.2. CD8^+^ T Cells

CD8^+^ CTL are essential for recognition and elimination of cells that are virally infected or display aberrant self. CTL recognize intracellular peptide bound to MHC class I on the cell surface, and upon activation they exert direct cytolytic effects against the target cell accompanied by the secretion of IFNγ. First evidence for the involvement of CTL came from Korbelik et al. in 1999. This group used an EMT6 mammary carcinoma model in which depletion of CD8^+^ T cells led to a 50% decrease in cures after PDT compared to unmanipulated mice [47]. Similar results were obtained by different groups using 2-iodo-5-ethylamino-9-diethylaminobenzo-phenotiazinium chloride or Photofrin^®^ as photosensitizers, albeit Photofrin® was used in a combination approach with 5-aza-2’-deoxycytidine to induce the tumor antigen P1A [53,54]. However, presence of P1A was not necessary to sustain long-term immunity. Likewise, adoptive transfer of CD8^+^ lymphocytes from cured animals was sufficient to protect naïve recipients from subsequent challenge with viable cancer cells from the same type [36,54]. It should be noted that this protection did not show a requirement for additional transfer of CD4^+^ T helper cells. A study by Saji et al. further substantiates these finding with an experiment where the transfer of CD8^+^ T cell-depleted splenocytes from PDT-cured mice into naïve recipients conferred protection to rechallenge [35]. Other studies frequently found elevated levels of CD8^+^ T cells after treatment, and closer examination of those cells revealed increased lytic activity against tumor cells in an antigen-specific manner [10,30,31]. On a clinical level, Abdel-Hady et al. were able to show that patients with vulval intraepithelial neoplasia who responded to PDT had increased levels of infiltrating CD8^+^ T cells after treatment [55].

### 3.3. Regulatory T Cells

Regulatory T cells comprise a unique subset among the CD4^+^ T cell subpopulations. Treg cells display regulatory and suppressive activity towards other immune cells, and especially effector T cells. On the molecular level they are characterized by the constitutive expression of high levels of the transmembrane protein CD25 (IL-2R α chain) [56] and cytotoxic T lymphocyte-associated antigen 4 (CTLA-4) [57] and stable expression of the lineage-specific transcription factor Foxp3 [58,59]. By now, it is well established that Treg are required for immunological tolerance and prevention of excessive inflammatory immune responses. Neonatal thymectomy in mice results in fatal T cell-mediated autoimmunity against various organs due to the lack of regulatory T cells [60]. Mutations in the X chromosome-encoded Foxp3 gene, as in human IPEX patients and Scurfy mice, leads to severe immune dysregulation, polyendocrinopathy, and enteropathy related to an inability to generate Treg and establish tolerance [59,61]. Their origin is either in the thymus (tTreg) in response to recognition of self-antigen during negative selection or in peripheral lymphoid organs (pTreg), where naïve T cells recognize antigens in a tolerogenic environment—like that of commensal bacteria in the gut or the cancer microenvironment—and differentiate into pTreg. tTreg are considered to provide tolerance towards self-antigens that are represented in the thymus, whereas the main function of pTreg is the establishment of tolerance to antigens that are either foreign but not harmful or self-antigens not presented in the thymus during T cell development [62,63]. In cancer it has been shown that Treg are the predominant T cell type accumulating in tumor tissue, and low T effector/Treg ratios correlate with poor prognosis in various tumor types [64,65,66]. Regulatory T cells employ various mechanisms to suppress effector T cells. Those mechanisms include sequestration of available IL-2 via the high affinity IL-2 receptor, CTLA-4 (CTL-associated protein 4)-mediated sequestration of CD80/86 on APC surfaces, production of inhibitory cytokines like IL-10 and IL-35, production of pericellular adenosine, and direct cytolysis of effector T cells by granzyme B and perforin [67]. In the tumor microenvironment, Treg keep effector T cells in an intermediate state via sequestration of IL-2 and production of TGF-β. Withdrawal of IL-2 prevents full activation of effector T cells and ensures continuous availability of IL-2 produced by partially activated T cells, which Treg need for their maintenance but do not produce themselves [68]. TGF-β prevents full cytotoxic effector differentiation of tumor-specific CD8^+^ T cells and keeps memory CD8^+^ T cells in an inactive state [69,70]. The importance of suppression of Treg to increase PDT efficiency has recently been shown in several studies [24,71]. Treg depletion by cyclophosphamide followed by PDT in mice bearing CT26 colon carcinomas led to improved long-term survival and development of memory immunity [24]. It should be mentioned that this need for Treg depletion constitutes sort of a conundrum: lymphocytes have been shown to be especially sensitive to PDT-mediated cell death (see below). Therefore, Treg residing in the tumor microenvironment should be depleted by PDT treatment, thus abolishing the need for external depletion. However, other studies using the same tumor model did not show this requirement. Although there have been substantial differences in the treatment protocols between those studies, the reason for this particular aspect has (to the best of our knowledge) not been addressed so far and clearly needs further elucidation. A recent study from Garg and coworkers looking at the brain immune contexture in a murine model of HGG showed a shift from Treg towards Th1/Th17/CTL following treatment with an ICD-based DC vaccine in a prophylactic as well as in a curative setting [14]. Zheng et al. found a decrease of Treg induction in vitro and in vivo in response to stimulation/treatment with DC which had been pulsed with PDT-treated Lewis lung carcinoma cells (LLC). This was accompanied by a significant inhibition of tumor growth upon challenge with live LLC cells [40]. In a clinical setting, blood analysis of patients with esophageal squamous cell carcinoma demonstrated that PDT with the PS Photofrin^®^ abolished the suppressive function of peripheral Treg, but had no effect on Treg numbers [72]. Taken together, these studies indicate effects of PDT on Treg, which have to be elucidated in order to harness the potential of PDT to break tumor-promoting immune suppression. 

## 4. Treatment Regimen and Modes of Cell Death

There are still considerable differences in treatment outcomes, limiting the successful translation of PDT into broad clinical application. These differences might be attributed to a variety of parameters including the PS and PS dose used, the drug–light interval, the applied fluence and fluence rate, and the light source itself. 

One determinant of the induction of antitumor immunity is the mode of cell death, which is triggered by the applied treatment regimen and can be influenced by any of the parameters mentioned above. An extensive review about the modes and mechanisms of cell death in PDT induced by different PS was published by Bacellar et al*.* in 2015 [73]. In general, PDT is able to result in apoptosis, necrosis, the so-called immunogenic cell death (ICD), and autophagy. Apoptosis is traditionally considered as a programmed form of cell death which is immunologically silent. The remnants of these “physiologically” dying cells are quickly removed afterwards by phagocytes. Therefore, cells undergoing apoptosis normally do not elicit a strong immune response or a detectable response at all, for that matter. In contrast to that, necrosis is the result of an insult or trauma that leads to rapid cell death. This form of cell death characteristically involves the uncontrolled release of cellular products and the initiation of an inflammatory response in the surrounding tissue, which can lead to severe bystander damage. Although an inflammatory response is necessary for the induction of the immune response, this process can be detrimental for the host when it cannot be resolved by the immune system. In light of these definitions, one would assume that necrotic cells or a mixture of necrotic and apoptotic cells should be more efficient in facilitating the development of a distinct immune response than apoptotic cells. Contrasting this, there are numerous reports demonstrating that apoptotic cells are superior to necrotic cells in inducing antitumor immunity [74,75,76,77,78,79,80]. Intensive research in the field of cell death in the following years led to the fairly new concept of ICD. ICD describes an immunogenic form of apoptosis or necrosis. Since the emergence of the concept of ICD it has been shown that tumor cells undergoing immunogenic apoptosis are more potent inducers of antitumor immune responses than cell dying via necrosis or nonimmunogenic apoptosis. Thus, it would be favorable to use and develop PSs which predominantly cause ICD in cancer cells for future approaches. Importantly, so far hypericin is the only PS shown to induce all major molecular and immunological hallmarks of ICD. This includes surface expression of CRT, HSP70, and HSP90 and secretion of ATP—four DAMPs crucial in ICD [80,81].

In an elegant study, Garg et al. demonstrated remarkable prophylactic and therapeutic success using DCs loaded with hypericin–PDT killed tumor cells in a model of HGG. In this case, the induction of antitumor immunity was dependent on the PDT-induced surface exposure of CRT and release of HMGB1 (amongst others). Notably, analysis of glioma cells treated with aminolevulinic acid (ALA)–PDT revealed no significant increase of those two DAMPs [14]. This clearly shows that the type of PS used can influence the determinants of cell death and its impact on subsequent antitumor immunity. Treatment parameters regarding the applied fluence and fluence rate also differ greatly among studies and have considerable effect on therapy outcome. It has been shown that at high-fluence PDT predominantly causes necrosis, while at medium to low fluences apoptosis or a mixture of apoptosis and necrosis is more prevalent [82]. Analysis of neutrophilia and secretion of cytokines (as a measure for inflammation) induced by different combinations of fluence and fluence rate by Henderson et al. showed that the most inflammatory response was triggered at a low fluence and a low fluence rate. However, the best tumor control was achieved with a high to intermediate fluence at a low fluence rate. This regimen only caused mild inflammation [19]. In a subsequent study, a sequential treatment approach consisting of a first session causing substantial inflammation (low fluence, low fluence rate) followed by a second session for tumor control (high/intermediate fluence, low fluence rate) yielded the best results with respect to control of the primary disease and induction of memory immunity [31]. These results are backed from findings by others indicating that low-dosed or vascular-targeted treatment regimens have better overall therapeutic outcome [36,43,48,83]. Additionally, clinical reports support a beneficial effect for therapy when using milder treatment protocols [45,84]. Another example for the impact of different treatment regimens and the influence they can have on the therapeutic approach comes from comparison of studies from Reginato et al. and Sanovic and coworkers: those two groups used the same tumor model (i.e., subcutaneously growing CT26 tumors in BALB/c mice) but their treatment regimen differed considerably in terms of PS (BPD vs hypericin) and in the fluence and fluence rate applied (120 J cm^−2^@100 mW cm^−2^ vs. 14 J cm^-2^@27 mW cm^−2^). Still, both groups achieved tumor cures and induction of memory immunity. However, in the system using BPD and a high light dose, depletion of Treg was necessary to induce tumor-specific immunity and unravel memory immunity [24,48]. 

A different aspect of the PS dose used is the effect on surrounding immune cells. T lymphocytes have been shown to be especially sensitive to PDT-induced death [85,86]. This might actually be beneficial therapy outcome since most of the T cells within the tumor microenvironment are considered to have an immunosuppressive phenotype. These T cells would be destroyed and the area would be repopulated by activated effector T cells. Indeed, it has been shown that shortly after PDT treatment, CD3^+^ lymphocytes (CD3 as part of the T cell receptor is expressed on all T cells) disappeared. About 24 h later, CD3^+^ T cells were present again with levels exceeding those prior to treatment [36,43]. Additionally, unpublished data from our group indicates that DCs show a higher susceptibility to PDT-mediated cell death in a dose-dependent manner with a variety of PSs compared to several different cancer cell lines tested. This raises the possibility that the PS dose used might affect antigen uptake and presentation (due to enhanced killing of antigen-presenting cells) necessary for launching the adaptive immune response.

## 5. Conclusions

The capability of PDT to effectively cure primary tumors and induce systemic and long-lasting immunity to combat metastases and recurring disease has been shown in numerous studies and a variety of settings over the past two decades. This holds the potential for this particular treatment modality to become a powerful therapeutic option for cancer patients. Recent years have seen a number of improvements to lift limitations given by the nature of PDT. Technical improvements include the development of new light sources to reach solid tumors that are neither superficial nor intraluminal. Furthermore, there are continuous efforts to develop PS which need less energy to be properly excited. This enables the use of light with longer wavelength, which penetrates deeper into tissue. Coupling of PS to antibodies specific for the tumor cells in question is supposed to improve targeting of the PS to the tumor. 

With increasing technical progress and improvements of PSs it should be possible to extend the applicability of PDT to a point where it would be a feasible therapy option for a broad spectrum of cancer entities. We believe that comparative analyses of the immunological effects elicited by different parameters of PDT (e.g., PS type and dose, fluence and fluence rate) would greatly enhance the understanding of the induction of antitumor immunity induced by this therapy. This would aid in developing protocols which efficiently fight off the primary tumor and boost the immune system to recognize and combat distant and recurring disease manifestation while being patient-sparing, easy to handle, and cost-effective.
